# Supplementary material for: Predicting Lifestyle from Positive Selection Data and Genome Properties in Oomycetes
Source: Pathogens. 2021 Jun 25;10(7):807. doi: 10.3390/pathogens10070807 (PMC8308905; doi:10.3390/pathogens10070807)
Supplement: Supplementary file 1 [file pathogens-10-00807-s001.zip › pathogens-1224178-supplementary.pdf]

pathogens

MDPI

Article

Predicting Lifestyle from Positive Selection Data and Genome Properties in Oomycetes

Daniel Gómez-Pérez [1], Eric Kemen [1,*]

[1] Center for Plant Molecular Biology (ZMBP), University of Tübingen, 72074 Tübingen, Germany; daniel.gomez-perez@uni-tuebingen.de

Supplementary Materials:

check for updates

Citation: Gómez-Pérez, D.; Kemen, E. Predicting Lifestyle from Positive Selection Data and Genome Properties in Oomycetes. *Pathogens* **2021**, *10*, 807. https://doi.org/10.3390/pathogens10070807

Academic Editor: Paolo Gonthier

Received: 30 April 2021

Accepted: 21 June 2021

Published: 25 June 2021

Publisher's Note: MDPI stays neutral with regard to jurisdictional claims in published maps and institutional affiliations.

Copyright: © 2021 by the authors. Licensee MDPI, Basel, Switzerland. This article is an open access article distributed under the terms and conditions of the Creative Commons Attribution (CC BY) license (https://creativecommons.org/licenses/by/4.0/).

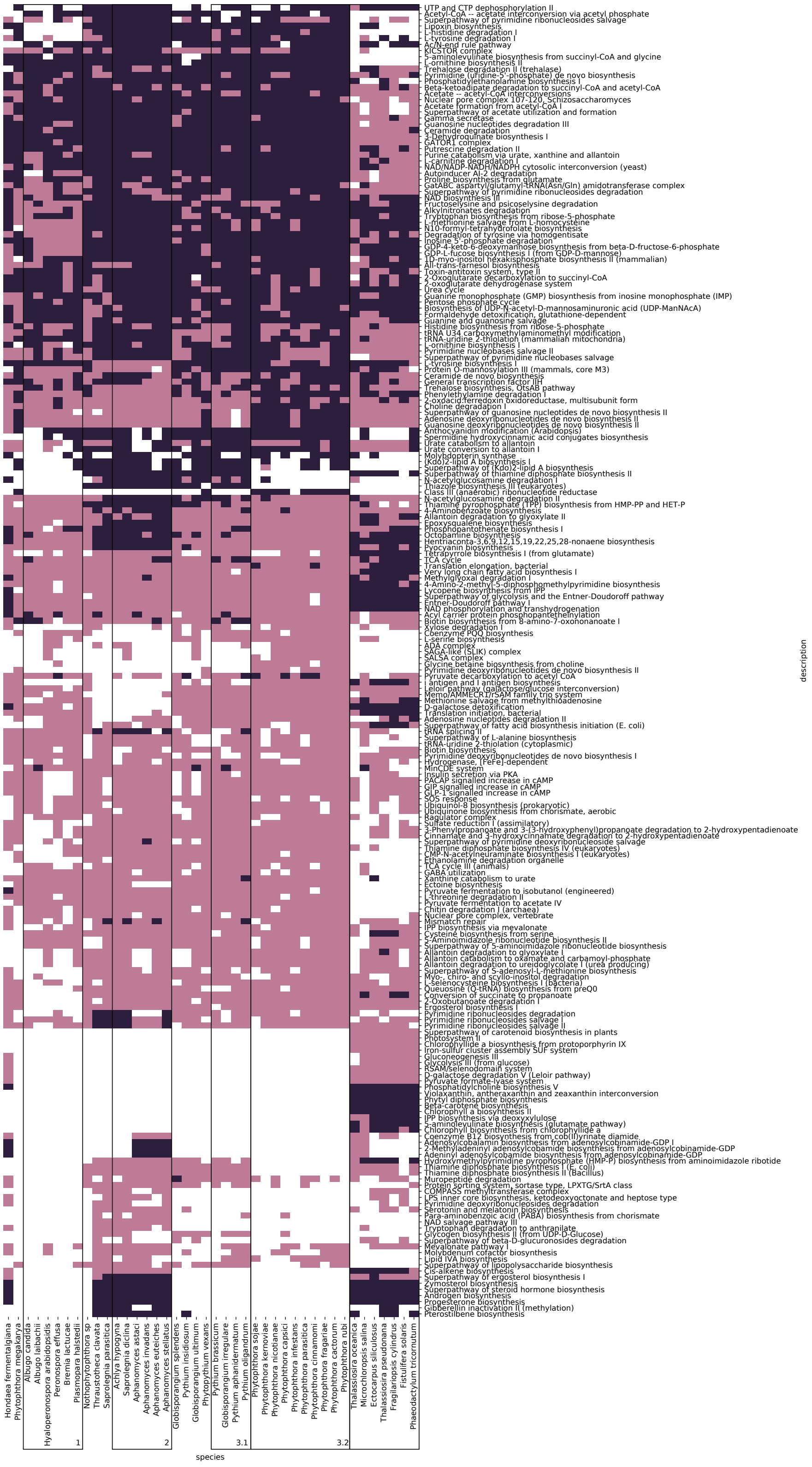

Figure S1. Differences in annotated cellular pathways from the stramenopile dataset. Shown are pathways which have up to 36 repeated values per taxa. The clusters from Figure 1 are encapsulated in a labeled square.

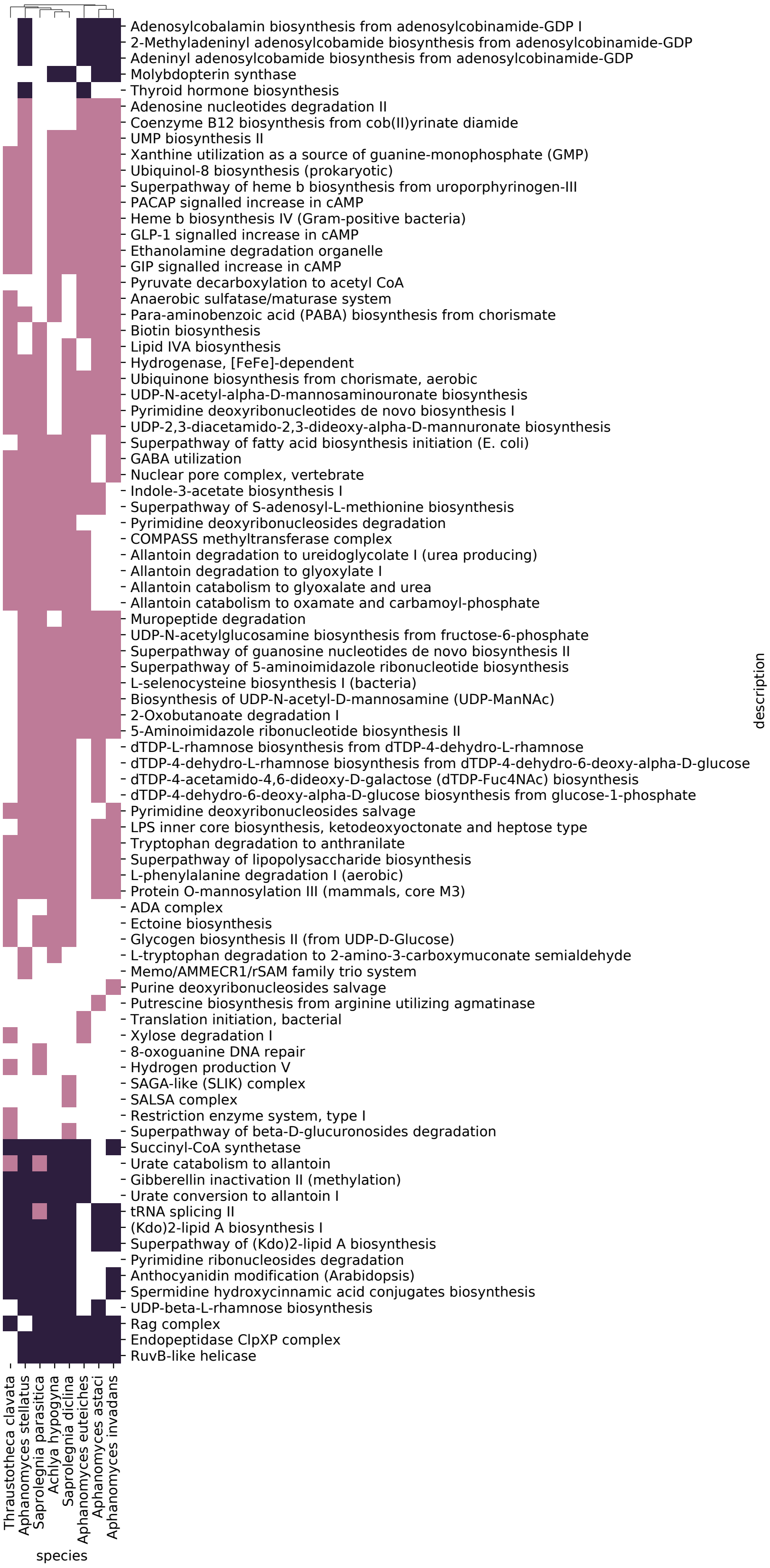

Figure S2. Differences in annotated cellular pathways for the members of the Saprolegniaceae family in the stramenopile dataset. Shown are pathways which are different in at least one taxa and have at least one complete loss in any of the taxa.

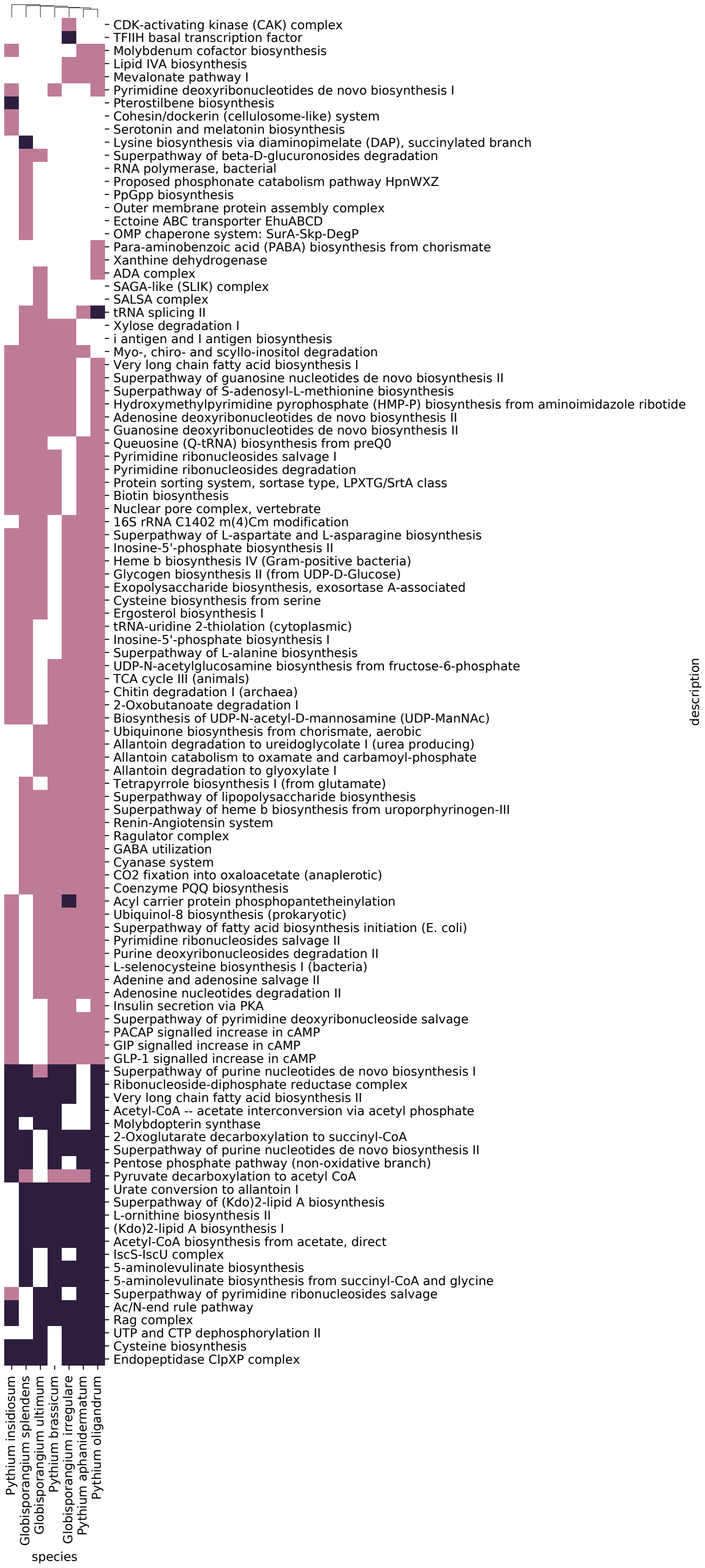

Figure S3. Differences in annotated cellular pathways for the members of the Pythiaceae family and *Globisporangium* genus in the stramenopile dataset. Shown are pathways which are different in at least one taxa and have at least one complete loss in any of the taxa.

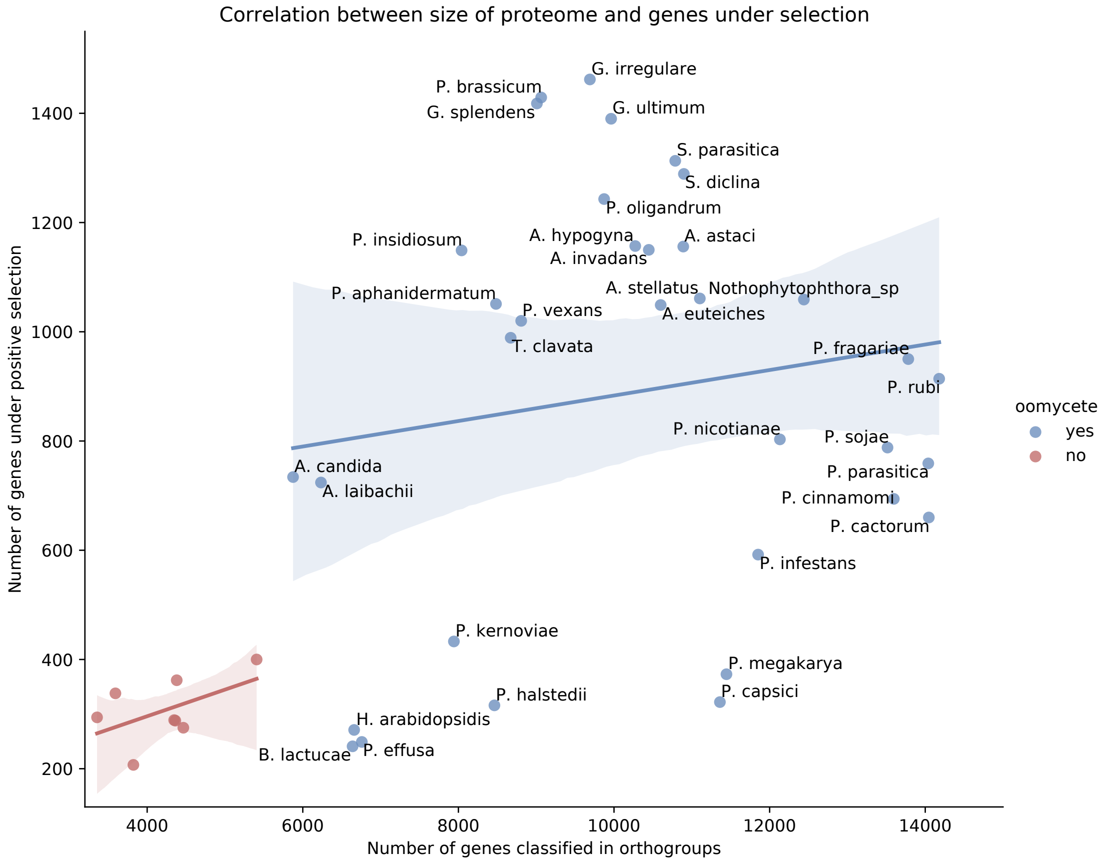

Figure S4. Correlation between genes under positive selection and proteome size in the stramenopile dataset. Oomycetes are in blue (Pearson's correlation, r = 0.15, *p* value = 0.39) and non-oomycetes in red (Pearson's correlation, r = 0.52, *p* value = 0.18). Pearson correlation represented as a straight line and the confidence interval represented as a lighter shade.

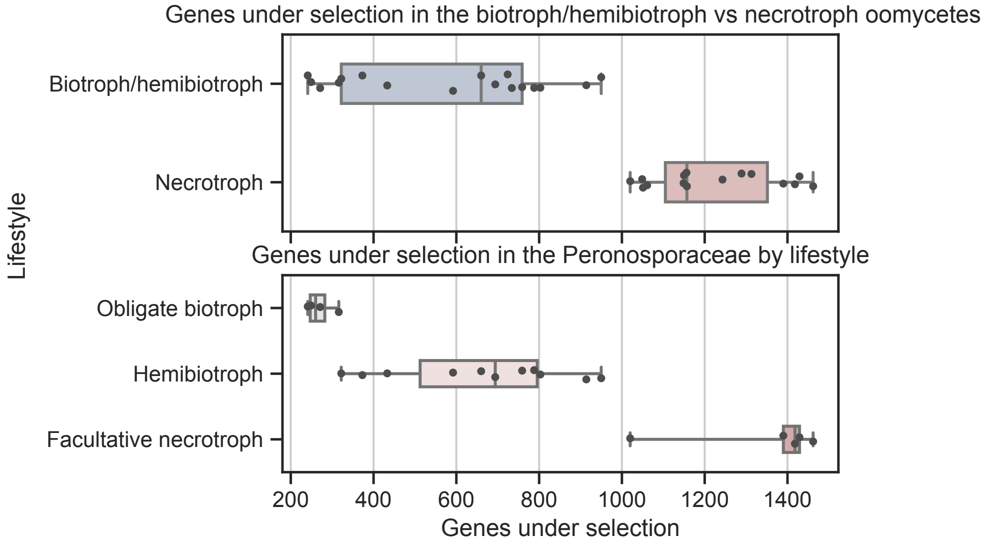

Figure S5. Comparison of the distribution of positive genes under selection for different lifestyles. Significance between the different categories is $p < 0.01$ in both the upper graph (Mann-Whitney test) and the lower graph (ANOVA one-tailed test).

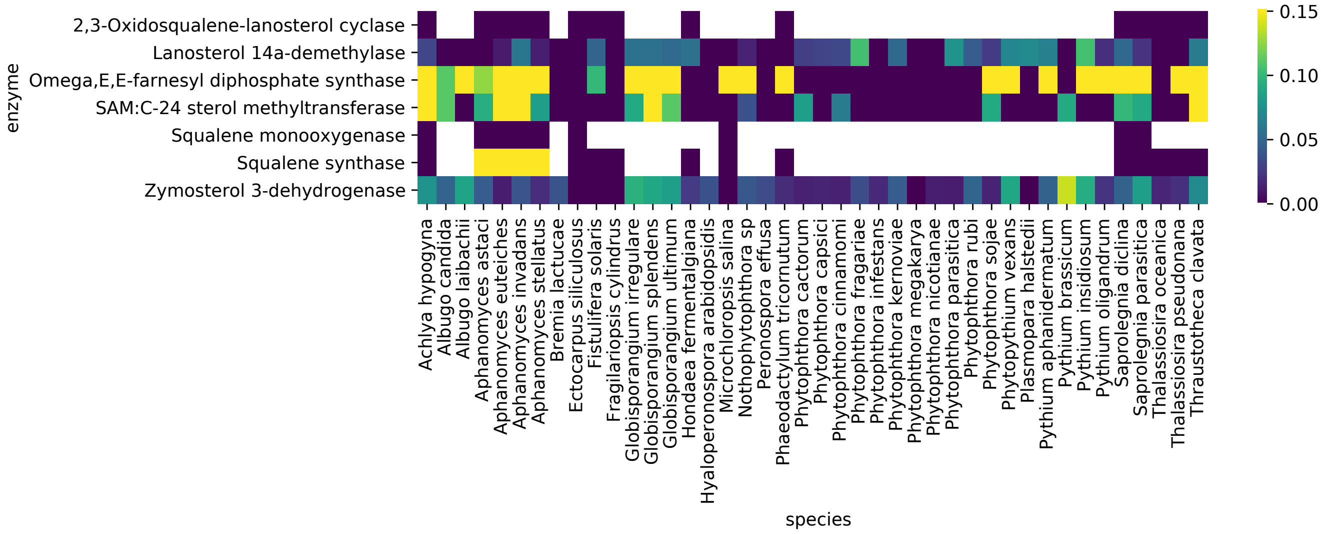

Figure S6. Sterol biosynthesis-related enzymes in stramenopiles. Heatmap of the presence and absence of the enzymes relating to sterol biosynthesis pathway in the stramenopiles. The yellow gradient represents the normalized ratio of predicted positive selection in genes with this annotation.

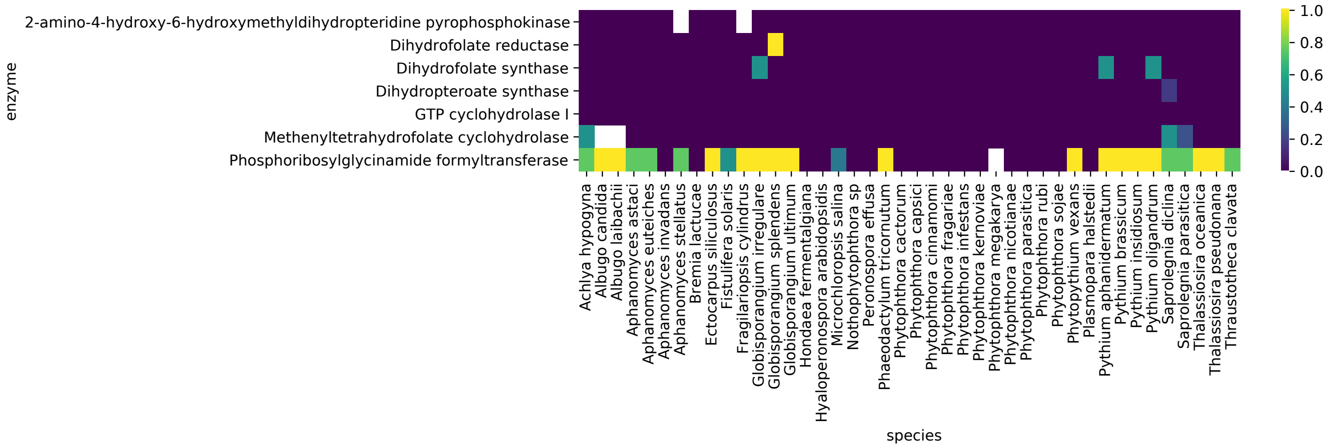

Figure S7. Tetrahydrofolate salvage and biosynthesis-related enzymes in stramenopiles. Heatmap of the presence and absence of the enzymes relating to tetrahydrofolate metabolism in the stramenopiles. The yellow gradient represents the ratio of predicted positive selection in genes with this annotation.

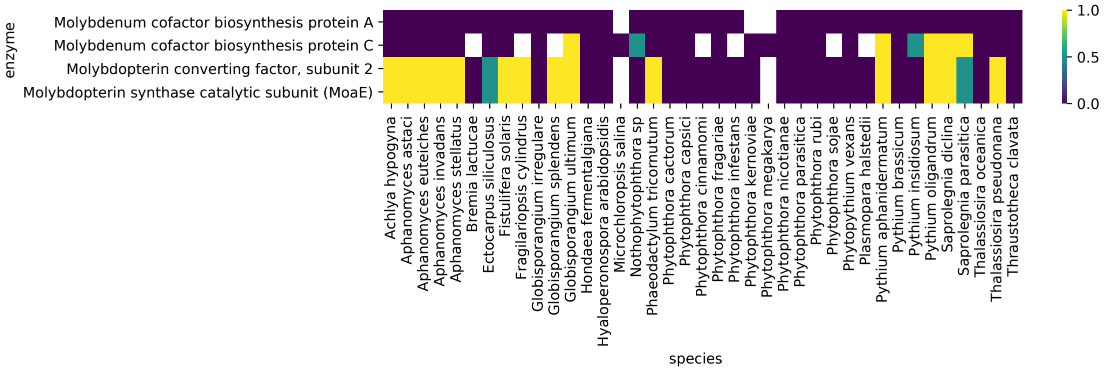

Figure S8. Molybdopterin biosynthesis-related enzymes in stramenopiles. Heatmap of the presence and absence of the enzymes relating to molybdopterin biosynthesis in the stramenopiles. The yellow gradient represents the ratio of predicted positive selection in genes with this annotation.

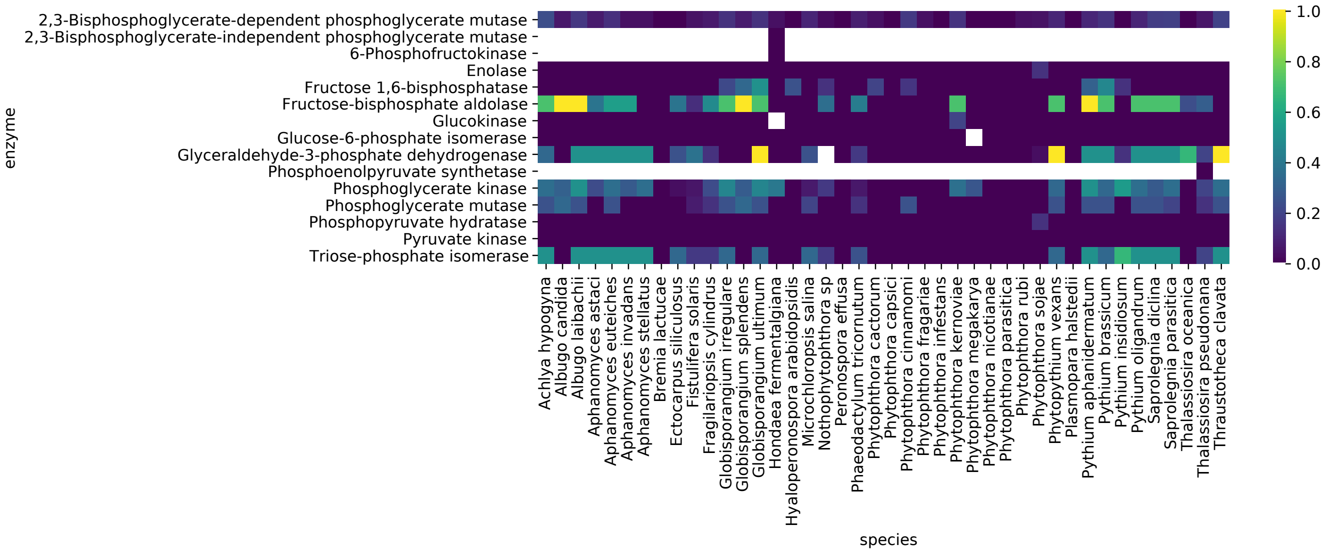

Figure S9. Glycolysis I, II and III-related enzymes in stramenopiles. Heatmap of the presence and absence of the enzymes relating to glycolysis pathway in the stramenopiles. The yellow gradient represents the normalized ratio of predicted positive selection in genes with this annotation.

Table S1. Summary of basidiomycete dataset.

Species name	Plant pathogen	Accession
Acaromyces ingoldii	no	GCA_003144295.1
Anthracocystis flocculosa	no	GCA_000417875.1
Apiotrichum porosum	no	GCA_003942205.1
Ceraceosorus bombacis	yes	GCA_900000165.1
Ceraceosorus guamensis	no	GCA_003144195.1
Ceratobasidium theobromae	yes	GCA_009078325.1
Cryptococcus amylolentus	no	GCA_001720205.1
Cryptococcus gattii	no	GCA_000855695.1
Cryptococcus neoformans	no	GCA_000149245.3
Cryptococcus wingfieldii	no	GCA_001720155.1
Cutaneotrichosporon oleaginosum	no	GCA_001027345.1
Fomitiporia mediterranea	yes	GCA_000271605.1
Jaapia argillacea	no	GCA_000697665.1
Jaminaea rosea	no	GCA_003144245.1
Kalmanozyma brasiliensis	no	GCA_000497045.1
Kockovaella imperatae	no	GCA_002102565.1
Kwoniella bestiolae	no	GCA_000512585.2
Kwoniella dejecticola	no	GCA_000512565.2
Kwoniella pini	no	GCA_000512605.2
Leucosporidium creatinivorum	no	GCA_002105055.1
Malassezia globosa	no	GCA_000181695.1
Malassezia restricta	no	GCA_003290485.1
Malassezia sympodialis	no	GCA_000349305.2
Meira miltonrushii	no	GCA_003144205.1
Melampsora larici-populina	yes	GCA_000204055.1
Microbotryum lychnidis-dioicae	yes	GCA_000166175.1
Mixia osmundae	yes	GCA_000708205.1
Moesziomyces antarcticus	no	GCA_000747765.1
Moesziomyces aphidis	no	GCA_000517465.1
Moniliophthora roreri	yes	GCA_001466705.1
Paxillus involutus	no	GCA_000827475.1
Peniophora sp	no	GCA_900536885.1
Piloderma croceum	no	GCA_000827315.1
Pseudomicrostroma glucosiphilum	no	GCA_003144135.1
Pseudozyma hubeiensis	no	GCA_000403515.1
Puccinia coronata	yes	GCA_002873125.1
Puccinia graminis	yes	GCA_000149925.1
Puccinia sorghi	yes	GCA_001263375.1
Puccinia striiformis	yes	GCA_002920065.1
Puccinia triticina	yes	GCA_000151525.2
Rhizoctonia solani	yes	GCA_000524645.1
Rhodotorula graminis	no	GCA_001329695.1
Rhodotorula toruloides	no	GCA_000320785.2
Saitozyma podzolica	no	GCA_003942215.1
Serendipita indica	no	GCA_000313545.1
Serendipita vermifera	no	GCA_000827415.1
Sporisorium graminicola	no	GCA_005498985.1
Sporisorium reilianum	yes	GCA_900162835.1
Sporisorium scitamineum	yes	GCA_001243155.1
Testicularia cyperi	yes	GCA_003144125.1
Tilletia controversa	yes	GCA_001645045.2
Tilletia laevis	yes	GCA_009428275.1
Tilletia walkeri	yes	GCA_009428295.1
Tilletiaria anomala	yes	GCA_000711695.1
Tilletiopsis washingtonensis	yes	GCA_003144115.1
Trichosporon asahii	no	GCA_000293215.1
Ustilago bromivora	yes	GCA_900080155.1
Ustilago hordei	yes	GCA_000286035.1
Ustilago maydis	yes	GCA_000328475.2
Ustilago trichophora	yes	GCA_900323505.1
Violaceomyces palustris	no	GCA_003144235.1
Wallemia hederae	no	GCA_004918325.1
Wallemia ichthyophaga	no	GCA_000400465.1
Wallemia mellicola	no	GCA_000263375.1
Xanthophyllomyces dendrorhous	no	GCA_001007165.2

Table S2. Summary of genomes used for the lifestyle model construction.

**Species name**	**Number of proteomes**	**Lifestyle**
Agaricus bisporus	1	S
Albugo candida and laibachii	2	B
Alternaria alternata, arborescens, gaisen and tenuissima	14	N
Aphanomyces euteiches and stellatus	2	N
Ascochyta rabiei	1	N
Aspergillus fumigatus, nidulans, oryzae and niger	34	S
Bipolaris maydis, oryzae, victoriae and zeicola	5	N
Bipolaris sorokiniana	2	H
Blumeria graminis	4	B
Botrytis cinerea	3	N
Bremia lactucae	1	B
Colletotrichum fioriniae, gloeosporioides, graminicola, higginsianum, incanum, nymphaeae, orbiculare, simmondsii and sublineola	14	H
Debaryomyces hansenii	1	S
Dothistroma septosporum	1	H
Erysiphe necator	1	B
Eutypa lata	1	N
Fusarium culmorum and graminearum	6	H
Gigaspora margarita	1	B
Globisporangium irregulare, splendens and ultimum	3	N
Gloeophyllum trabeum	1	S
Hyaloperonospora arabidopsidis	1	B
Komagataella phaffii	5	S
Leptosphaeria maculans	1	H
Macrophomina phaseolina	1	H
Marssonina brunnea	1	H
Melampsora laricis-populina	1	B
Microbotryum violaceum	1	B
Monilinia laxa	1	N
Moniliophthora perniciosa and roreri	3	H
Neurospora crassa	2	S
Oidium neolycopersici	1	B
Parastagonospora nodorum	1	N
Peronospora effusa	2	B
Phytophthora cactorum, effusa, fragariae, infestans, kernoviae, megakarya, nicotianae, palmivora, parasitica, ramorum, rubi and sojae	38	H
Plasmodiophora brassicae	2	B
Plasmopara halstedii	1	B
Pleurotus ostreatu	1	S
Pseudocercospora fijiensis	1	H
Puccinia coronata, graminis, sorghi, striiformis and triticina	10	B
Pyrenophora teres f teres and tritici-repentis	18	N
Pyricularia oryzae	4	H
Pythium aphanidermatum and brassicum	2	N
Ramularia collo-cygni	1	H
Rhizoctonia solani	7	N
Rhizopus delemar	1	S
Saccharomyces cerevisiae	60	S
Schizosaccharomyces pombe	1	S
Sclerotinia borealis and sclerotiorum	3	N
Serpula lacrymans	2	S
Setosphaeria turcica	1	H
Sphaerobolus stellatus	1	S
Sporisorium reilianum	2	B
Stereum hirsutum	1	S
Synchytrium endobioticum	2	B
Taphrina deformans	1	B
Thraustotheca clavata	1	S
Tilletia indica	3	H
Tilletiaria anomala	1	B
Trametes versicolor	1	S
Tremella mesenterica	2	B
Trichoderma harzianum, reesei and virens	7	S
Uncinocarpus reesii	1	S
Ustilaginoidea virens	2	B
Ustilago bromivora, hordei and maydis	3	B
Venturia inaequalis	4	H
Verticillium dahliae	10	H
Yarrowia lipolytica	13	S
Zymoseptoria brevis and tritici	6	H

S: saprotroph, N: necrotroph, H: hemibiotroph, B: biotroph

Table S3. Significant GO terms with a depth higher than 7 found enriched in the positively selected proteins in plant fungal pathogens.

GO number	Name	Ratio in study	Ratio in population	Depth	−log10 of *p* value
GO:0009064	glutamine family amino acid metabolic process	140/13729	458/237259	8	57.33
GO:0006165	nucleoside diphosphate phosphorylation	99/13729	320/237259	8	40.37
GO:0006096	glycolytic process	80/13729	266/237259	12	31.2
GO:0006399	tRNA metabolic process	239/13729	1881/237259	8	25.41
GO:1901607	alpha-amino acid biosynthetic process	138/13729	830/237259	8	24.68
GO:0006525	arginine metabolic process	54/13729	157/237259	9	23.51
GO:0006546	glycine catabolic process	40/13729	86/237259	10	22.7
GO:0001510	RNA methylation	56/13729	211/237259	8	18.2
GO:0006750	glutathione biosynthetic process	29/13729	56/237259	8	17.46
GO:0034470	ncRNA processing	186/13729	1549/237259	8	16.55
GO:0008033	tRNA processing	130/13729	991/237259	9	13.87
GO:1901606	alpha-amino acid catabolic process	62/13729	359/237259	8	10.58
GO:0006418	tRNA aminoacylation for protein translation	109/13729	880/237259	10	9.59
GO:0009435	NAD biosynthetic process	34/13729	145/237259	11	8.46
GO:0016579	protein deubiquitination	59/13729	393/237259	9	7.36
GO:0009150	purine ribonucleotide metabolic process	119/13729	1092/237259	9	6.92
GO:0015693	magnesium ion transport	28/13729	123/237259	8	6.23
GO:0006633	fatty acid biosynthetic process	35/13729	219/237259	8	4.08
GO:0015031	protein transport	179/13729	2051/237259	8	3.95
GO:0006355	regulation of transcription, DNA-templated	337/13729	4376/237259	9	3.6
GO:0009165	nucleotide biosynthetic process	123/13729	1368/237259	8	2.48
GO:0006605	protein targeting	38/13729	288/237259	10	2.38
GO:0001522	pseudouridine synthesis	35/13729	260/237259	8	2.28
GO:0006364	rRNA processing	57/13729	515/237259	9	2.22
GO:0006511	ubiquitin-dependent protein catabolic process	86/13729	934/237259	8	1.37

Table S4. Significantly enriched terms relating to biological processes in the positively selected obligate biotroph proteins.

GO number	Name	Ratio in study	Ratio in population	Depth	−log10 of *p* value
GO:2000113	negative regulation of cellular macromolecule biosynthetic process	12/2535	54/75580	7	3.44
GO:0043648	dicarboxylic acid metabolic process	16/2535	77/75580	6	3.3
GO:0051253	negative regulation of RNA metabolic process	11/2535	47/75580	7	3.18
GO:0031324	negative regulation of cellular metabolic process	14/2535	70/75580	5	2.98
GO:0008033	tRNA processing	33/2535	289/75580	9	2.89
GO:0016053	organic acid biosynthetic process	38/2535	453/75580	4	2.69
GO:0018193	peptidyl-amino acid modification	29/2535	315/75580	7	2.63
GO:0010605	negative regulation of macromolecule metabolic process	21/2535	172/75580	5	2.52
GO:0006399	tRNA metabolic process	49/2535	597/75580	8	2.49
GO:0009064	glutamine family amino acid metabolic process	16/2535	118/75580	8	2.35
GO:0007018	microtubule-based movement	35/2535	407/75580	3	2.35
GO:0006082	organic acid metabolic process	79/2535	1249/75580	3	2.34
GO:0006396	RNA processing	80/2535	1217/75580	7	2.3
GO:0006468	protein phosphorylation	97/2535	1582/75580	7	2.26
GO:0034637	cellular carbohydrate biosynthetic process	16/2535	120/75580	5	2.26
GO:0016310	phosphorylation	110/2535	1819/75580	5	2.17
GO:0043412	macromolecule modification	206/2535	3434/75580	4	2.13
GO:0019752	carboxylic acid metabolic process	72/2535	1134/75580	5	2.13
GO:0060255	regulation of macromolecule metabolic process	67/2535	1054/75580	4	2.13
GO:0007017	microtubule-based process	41/2535	556/75580	2	2.1
GO:0006725	cellular aromatic compound metabolic process	212/2535	4200/75580	3	2.08
GO:1901360	organic cyclic compound metabolic process	220/2535	4285/75580	3	2.08
GO:0006464	cellular protein modification process	178/2535	3032/75580	6	2.08
GO:0009058	biosynthetic process	168/2535	3331/75580	2	2.07
GO:0044267	cellular protein metabolic process	208/2535	3721/75580	5	2.07
GO:0046483	heterocycle metabolic process	215/2535	4214/75580	3	2.07
GO:0034470	ncRNA processing	41/2535	560/75580	8	2.05
GO:0034660	ncRNA metabolic process	57/2535	880/75580	7	2.05
GO:0090304	nucleic acid metabolic process	168/2535	3199/75580	5	2.05
GO:0019538	protein metabolic process	245/2535	4869/75580	4	2.04
GO:0050789	regulation of biological process	111/2535	2041/75580	2	2.04
GO:0044249	cellular biosynthetic process	151/2535	3012/75580	3	2.01
GO:0006139	nucleobase-containing compound metabolic process	192/2535	3938/75580	4	2.0
GO:0016070	RNA metabolic process	122/2535	2310/75580	6	1.97
GO:0044260	cellular macromolecule metabolic process	302/2535	5672/75580	4	1.95
GO:1901566	organonitrogen compound biosynthetic process	91/2535	1648/75580	4	1.94
GO:0034641	cellular nitrogen compound metabolic process	245/2535	4998/75580	3	1.93
GO:1901564	organonitrogen compound metabolic process	340/2535	6708/75580	3	1.92
GO:0043170	macromolecule metabolic process	432/2535	8280/75580	3	1.89
GO:0006807	nitrogen compound metabolic process	500/2535	9762/75580	2	1.87
GO:0044237	cellular metabolic process	541/2535	10414/75580	2	1.86
GO:0071704	organic substance metabolic process	610/2535	11484/75580	2	1.84
GO:0044238	primary metabolic process	554/2535	10641/75580	2	1.84
GO:0008152	metabolic process	643/2535	12234/75580	1	1.83
GO:0046394	carboxylic acid biosynthetic process	31/2535	376/75580	6	1.83
GO:0031323	regulation of cellular metabolic process	58/2535	932/75580	4	1.77
GO:0045892	negative regulation of transcription, DNA-templated	9/2535	43/75580	10	1.69
GO:2000112	regulation of cellular macromolecule biosynthetic process	52/2535	816/75580	6	1.59
GO:0010468	regulation of gene expression	58/2535	936/75580	5	1.58
GO:0065007	biological regulation	113/2535	2204/75580	1	1.57
GO:1901576	organic substance biosynthetic process	150/2535	3124/75580	3	1.54
GO:0005975	carbohydrate metabolic process	49/2535	755/75580	3	1.54
GO:0050794	regulation of cellular process	99/2535	1887/75580	3	1.48
GO:0009086	methionine biosynthetic process	7/2535	26/75580	10	1.47
GO:0006520	cellular amino acid metabolic process	51/2535	803/75580	6	1.47
GO:0080090	regulation of primary metabolic process	57/2535	927/75580	4	1.44
GO:0044283	small molecule biosynthetic process	45/2535	674/75580	3	1.42
GO:0008652	cellular amino acid biosynthetic process	25/2535	291/75580	7	1.41
GO:1901605	alpha-amino acid metabolic process	29/2535	364/75580	7	1.35

Table S5. Significantly enriched terms relating to biological processes in the positively selected hemibiotroph proteins.

GO number	Name	Ratio in study	Ratio in population	Depth	−log10 of *p* value
GO:0009086	methionine biosynthetic process	13/6255	64/222540	10	3.38
GO:0051274	beta-glucan biosynthetic process	18/6255	104/222540	8	3.31
GO:0051253	negative regulation of RNA metabolic process	16/6255	91/222540	7	3.26
GO:0043648	dicarboxylic acid metabolic process	20/6255	150/222540	6	3.25
GO:0009082	branched-chain amino acid biosynthetic process	18/6255	81/222540	5	3.06
GO:0010605	negative regulation of macromolecule metabolic process	34/6255	300/222540	5	2.96
GO:0034637	cellular carbohydrate biosynthetic process	29/6255	226/222540	5	2.94
GO:0031324	negative regulation of cellular metabolic process	20/6255	129/222540	5	2.79
GO:0009312	oligosaccharide biosynthetic process	19/6255	146/222540	5	2.74
GO:2000113	negative regulation of cellular macromolecule biosynthetic process	15/6255	101/222540	7	2.7
GO:0044262	cellular carbohydrate metabolic process	35/6255	455/222540	4	2.67
GO:0016051	carbohydrate biosynthetic process	37/6255	333/222540	4	2.61
GO:0000097	sulfur amino acid biosynthetic process	15/6255	121/222540	5	2.46
GO:0007017	microtubule-based process	72/6255	1355/222540	2	2.39
GO:0006355	regulation of transcription, DNA-templated	75/6255	1176/222540	9	2.39
GO:2000112	regulation of cellular macromolecule biosynthetic process	84/6255	1307/222540	6	2.39
GO:0051273	beta-glucan metabolic process	19/6255	190/222540	7	2.37
GO:0006396	RNA processing	95/6255	1887/222540	7	2.33
GO:0016071	mRNA metabolic process	52/6255	831/222540	7	2.32
GO:0051171	regulation of nitrogen compound metabolic process	95/6255	1522/222540	4	2.31
GO:0006974	cellular response to DNA damage stimulus	68/6255	1193/222540	4	2.29
GO:0010468	regulation of gene expression	104/6255	1526/222540	5	2.28
GO:0006950	response to stress	88/6255	1528/222540	2	2.27
GO:0006397	mRNA processing	42/6255	664/222540	8	2.27
GO:0019222	regulation of metabolic process	118/6255	1794/222540	3	2.27
GO:0051252	regulation of RNA metabolic process	81/6255	1206/222540	6	2.26
GO:0060255	regulation of macromolecule metabolic process	117/6255	1749/222540	4	2.25
GO:0033554	cellular response to stress	72/6255	1253/222540	3	2.24
GO:0051716	cellular response to stimulus	73/6255	1254/222540	2	2.24
GO:0050896	response to stimulus	96/6255	1605/222540	1	2.22
GO:0031323	regulation of cellular metabolic process	100/6255	1555/222540	4	2.21
GO:0005975	carbohydrate metabolic process	156/6255	2565/222540	3	2.19
GO:0050789	regulation of biological process	172/6255	3715/222540	2	2.12
GO:0048519	negative regulation of biological process	35/6255	527/222540	3	2.11
GO:0050794	regulation of cellular process	154/6255	3447/222540	3	2.05
GO:0044249	cellular biosynthetic process	208/6255	5071/222540	3	2.01
GO:0065007	biological regulation	178/6255	4153/222540	1	2.01
GO:0016070	RNA metabolic process	148/6255	3483/222540	6	2.01
GO:0006468	protein phosphorylation	215/6255	4156/222540	7	2.0
GO:0016310	phosphorylation	235/6255	4699/222540	5	2.0
GO:0009058	biosynthetic process	230/6255	5732/222540	2	1.96
GO:0006793	phosphorus metabolic process	292/6255	6981/222540	3	1.94
GO:0043412	macromolecule modification	366/6255	7831/222540	4	1.94
GO:0006464	cellular protein modification process	327/6255	7218/222540	6	1.94
GO:0044267	cellular protein metabolic process	372/6255	8250/222540	5	1.92
GO:0006796	phosphate-containing compound metabolic process	291/6255	6936/222540	4	1.9
GO:0019538	protein metabolic process	459/6255	11568/222540	4	1.84
GO:0034645	cellular macromolecule biosynthetic process	92/6255	1977/222540	5	1.82
GO:1901576	organic substance biosynthetic process	209/6255	5300/222540	3	1.82
GO:0044260	cellular macromolecule metabolic process	524/6255	13650/222540	4	1.81
GO:1901564	organonitrogen compound metabolic process	558/6255	15181/222540	3	1.79
GO:0043170	macromolecule metabolic process	713/6255	19213/222540	3	1.74
GO:0044237	cellular metabolic process	841/6255	22391/222540	2	1.71
GO:0006807	nitrogen compound metabolic process	788/6255	22412/222540	2	1.7
GO:0044238	primary metabolic process	941/6255	25043/222540	2	1.69
GO:0071704	organic substance metabolic process	1007/6255	26763/222540	2	1.67
GO:0008152	metabolic process	1069/6255	28228/222540	1	1.67
GO:0055085	transmembrane transport	202/6255	5180/222540	4	1.66
GO:0006813	potassium ion transport	25/6255	336/222540	6	1.6
GO:0045892	negative regulation of transcription, DNA-templated	11/6255	82/222540	10	1.43
GO:0009059	macromolecule biosynthetic process	99/6255	2252/222540	4	1.39

Table S6. Enriched terms relating to biological processes in the positively selected plant necrotrophs.

GO number	Name	Ratio in study	Ratio in population	Depth	−log10 of *p* value
GO:0006190	inosine salvage	8/9880	8/129511	11	3.63
GO:0009088	threonine biosynthetic process	8/9880	8/129511	10	3.63
GO:0006425	glutaminyl-tRNA aminoacylation	8/9880	8/129511	11	3.63
GO:1901031	regulation of response to reactive oxygen species	9/9880	11/129511	6	3.5
GO:0046168	glycerol-3-phosphate catabolic process	13/9880	18/129511	7	3.47
GO:0072350	tricarboxylic acid metabolic process	17/9880	32/129511	6	3.28
GO:0006537	glutamate biosynthetic process	14/9880	15/129511	10	3.23
GO:0006166	purine ribonucleoside salvage	14/9880	23/129511	10	3.22
GO:0009084	glutamine family amino acid biosynthetic process	27/9880	86/129511	9	3.14
GO:0005992	trehalose biosynthetic process	26/9880	73/129511	7	3.13
GO:0009082	branched-chain amino acid biosynthetic process	31/9880	67/129511	5	3.1
GO:0060271	cilium assembly	30/9880	122/129511	7	3.06
GO:0009064	glutamine family amino acid metabolic process	47/9880	153/129511	8	2.99
GO:0000096	sulfur amino acid metabolic process	28/9880	123/129511	4	2.98
GO:0006144	purine nucleobase metabolic process	18/9880	50/129511	7	2.97
GO:0006555	methionine metabolic process	19/9880	56/129511	9	2.96
GO:0006536	glutamate metabolic process	22/9880	39/129511	9	2.96
GO:0033014	tetrapyrrole biosynthetic process	29/9880	109/129511	5	2.95
GO:0051274	beta-glucan biosynthetic process	29/9880	75/129511	8	2.93
GO:0016573	histone acetylation	22/9880	75/129511	11	2.93
GO:0003341	cilium movement	25/9880	75/129511	4	2.93
GO:0001522	pseudouridine synthesis	45/9880	154/129511	8	2.93
GO:0071897	DNA biosynthetic process	18/9880	34/129511	7	2.92
GO:0006816	calcium ion transport	27/9880	124/129511	7	2.91
GO:0006102	isocitrate metabolic process	9/9880	16/129511	7	2.91
GO:0000097	sulfur amino acid biosynthetic process	21/9880	82/129511	5	2.89
GO:0032012	regulation of ARF protein signal transduction	16/9880	45/129511	9	2.88
GO:0030488	tRNA methylation	17/9880	51/129511	11	2.85
GO:0007154	cell communication	28/9880	76/129511	2	2.83
GO:0018205	peptidyl-lysine modification	32/9880	148/129511	8	2.82
GO:0017038	protein import	33/9880	133/129511	9	2.8
GO:0006606	protein import into nucleus	25/9880	83/129511	10	2.8
GO:0051056	regulation of small GTPase mediated signal transduction	28/9880	70/129511	7	2.78
GO:0006414	translational elongation	28/9880	90/129511	6	2.78
GO:0006075	(1->3)-beta-D-glucan biosynthetic process	22/9880	64/129511	9	2.75
GO:0009086	methionine biosynthetic process	18/9880	46/129511	10	2.74
GO:0034637	cellular carbohydrate biosynthetic process	56/9880	165/129511	5	2.73
GO:0006525	arginine metabolic process	19/9880	58/129511	9	2.72
GO:0016051	carbohydrate biosynthetic process	61/9880	238/129511	4	2.72
GO:0001510	RNA methylation	43/9880	205/129511	8	2.72
GO:0006400	tRNA modification	55/9880	228/129511	10	2.71
GO:0009250	glucan biosynthetic process	30/9880	84/129511	7	2.71
GO:0051273	beta-glucan metabolic process	30/9880	150/129511	7	2.69
GO:0006096	glycolytic process	41/9880	150/129511	12	2.69
GO:2000113	negative regulation of cellular macromolecule biosynthetic process	20/9880	71/129511	7	2.69
GO:0070925	organelle assembly	41/9880	197/129511	5	2.68
GO:0043648	dicarboxylic acid metabolic process	34/9880	120/129511	6	2.67
GO:0043547	positive regulation of GTPase activity	15/9880	21/129511	6	2.67
GO:0010605	negative regulation of macromolecule metabolic process	52/9880	240/129511	5	2.64
GO:0009312	oligosaccharide biosynthetic process	31/9880	106/129511	5	2.63
GO:0006073	cellular glucan metabolic process	31/9880	159/129511	6	2.62
GO:0016570	histone modification	39/9880	199/129511	7	2.6
GO:1901615	organic hydroxy compound metabolic process	35/9880	190/129511	3	2.59
GO:0006091	generation of precursor metabolites and energy	57/9880	294/129511	3	2.55
GO:0006401	RNA catabolic process	38/9880	181/129511	7	2.53
GO:0072330	monocarboxylic acid biosynthetic process	28/9880	138/129511	7	2.48
GO:1901607	alpha-amino acid biosynthetic process	57/9880	356/129511	8	2.47
GO:0006418	tRNA aminoacylation for protein translation	56/9880	328/129511	10	2.46
GO:0048583	regulation of response to stimulus	51/9880	300/129511	3	2.46
GO:0016052	carbohydrate catabolic process	51/9880	261/129511	4	2.45
GO:0032259	methylation	72/9880	386/129511	2	2.45
GO:0008033	tRNA processing	96/9880	403/129511	9	2.45
GO:0034472	snRNA 3′-end processing	10/9880	22/129511	10	2.45
GO:0072594	establishment of protein localization to organelle	43/9880	251/129511	6	2.44
GO:0006457	protein folding	60/9880	404/129511	2	2.43
GO:0044262	cellular carbohydrate metabolic process	66/9880	330/129511	4	2.43
GO:0046034	ATP metabolic process	43/9880	227/129511	2	2.43
GO:0072521	purine-containing compound metabolic process	74/9880	488/129511	4	2.42
GO:0031324	negative regulation of cellular metabolic process	22/9880	95/129511	5	2.37
GO:0043414	macromolecule methylation	52/9880	320/129511	5	2.34
GO:0008652	cellular amino acid biosynthetic process	79/9880	427/129511	7	2.33
GO:0032787	monocarboxylic acid metabolic process	81/9880	463/129511	6	2.32
GO:0034470	ncRNA processing	140/9880	735/129511	8	2.3
GO:0006813	potassium ion transport	49/9880	309/129511	6	2.29
GO:0018193	peptidyl-amino acid modification	85/9880	518/129511	7	2.28
GO:0046394	carboxylic acid biosynthetic process	107/9880	573/129511	6	2.28
GO:1901605	alpha-amino acid metabolic process	87/9880	557/129511	7	2.26
GO:0009451	RNA modification	120/9880	540/129511	7	2.26
GO:0016053	organic acid biosynthetic process	144/9880	682/129511	4	2.23
GO:0006310	DNA recombination	40/9880	233/129511	7	2.22
GO:0034660	ncRNA metabolic process	201/9880	1090/129511	7	2.22
GO:0034613	cellular protein localization	44/9880	274/129511	4	2.22
GO:0006399	tRNA metabolic process	157/9880	748/129511	8	2.21
GO:0007018	microtubule-based movement	116/9880	839/129511	3	2.21
GO:0006814	sodium ion transport	17/9880	63/129511	6	2.19
GO:0006520	cellular amino acid metabolic process	167/9880	1097/129511	6	2.19
GO:0006355	regulation of transcription, DNA-templated	145/9880	1045/129511	9	2.18
GO:0044283	small molecule biosynthetic process	179/9880	1074/129511	3	2.18
GO:0006812	cation transport	162/9880	1214/129511	5	2.17
GO:0007017	microtubule-based process	141/9880	1103/129511	2	2.16
GO:0080090	regulation of primary metabolic process	174/9880	1307/129511	4	2.15
GO:0010468	regulation of gene expression	183/9880	1307/129511	5	2.15
GO:1901575	organic substance catabolic process	189/9880	1657/129511	3	2.15
GO:0051252	regulation of RNA metabolic process	147/9880	1056/129511	6	2.14
GO:0006629	lipid metabolic process	197/9880	1627/129511	3	2.14
GO:0000398	mRNA splicing, via spliceosome	50/9880	321/129511	11	2.12
GO:0005975	carbohydrate metabolic process	200/9880	1738/129511	3	2.11
GO:0006066	alcohol metabolic process	25/9880	121/129511	4	2.1
GO:0006082	organic acid metabolic process	321/9880	1814/129511	3	2.1
GO:0006396	RNA processing	263/9880	1641/129511	7	2.1
GO:0031323	regulation of cellular metabolic process	177/9880	1325/129511	4	2.09

Table S7. Enriched terms relating to biological processes in the positively selected plant necrotrophs (continued).

GO number	Name	Ratio in study	Ratio in population	Depth	−log10 of *p* value
GO:2000112	regulation of cellular macromolecule biosynthetic process	157/9880	1151/129511	6	2.08
GO:0005976	polysaccharide metabolic process	32/9880	175/129511	4	2.08
GO:0019222	regulation of metabolic process	215/9880	1513/129511	3	2.08
GO:0060255	regulation of macromolecule metabolic process	210/9880	1484/129511	4	2.07
GO:0031047	gene silencing by RNA	8/9880	15/129511	8	2.07
GO:0034645	cellular macromolecule biosynthetic process	217/9880	1621/129511	5	2.06
GO:0043604	amide biosynthetic process	114/9880	946/129511	5	2.04
GO:0016043	cellular component organization	226/9880	2030/129511	3	2.02
GO:0016070	RNA metabolic process	453/9880	2964/129511	6	2.01
GO:0009056	catabolic process	208/9880	1779/129511	2	2.0
GO:0050896	response to stimulus	141/9880	1228/129511	1	2.0
GO:0043087	regulation of GTPase activity	17/9880	65/129511	5	2.0
GO:0071840	cellular component organization or biogenesis	233/9880	2151/129511	2	2.0
GO:0009059	macromolecule biosynthetic process	231/9880	1856/129511	4	1.99
GO:0022607	cellular component assembly	110/9880	906/129511	4	1.99
GO:0019752	carboxylic acid metabolic process	270/9880	1645/129511	5	1.99
GO:1901566	organonitrogen compound biosynthetic process	319/9880	2320/129511	4	1.99
GO:0050789	regulation of biological process	385/9880	3249/129511	2	1.94
GO:0009112	nucleobase metabolic process	24/9880	116/129511	6	1.93
GO:0044281	small molecule metabolic process	405/9880	3119/129511	2	1.92
GO:0006885	regulation of pH	15/9880	53/129511	8	1.91
GO:0065007	biological regulation	409/9880	3527/129511	1	1.91
GO:0050794	regulation of cellular process	337/9880	3032/129511	3	1.91
GO:0046148	pigment biosynthetic process	17/9880	66/129511	3	1.91
GO:0050790	regulation of catalytic activity	38/9880	227/129511	3	1.88
GO:0006811	ion transport	328/9880	3267/129511	4	1.85
GO:0044249	cellular biosynthetic process	543/9880	4181/129511	3	1.85
GO:0090304	nucleic acid metabolic process	569/9880	5033/129511	5	1.84
GO:1901576	organic substance biosynthetic process	564/9880	4428/129511	3	1.83
GO:0006464	cellular protein modification process	551/9880	5511/129511	6	1.82
GO:0009058	biosynthetic process	613/9880	4761/129511	2	1.81
GO:0006793	phosphorus metabolic process	512/9880	5401/129511	3	1.8
GO:0006419	alanyl-tRNA aminoacylation	8/9880	16/129511	11	1.8
GO:0006101	citrate metabolic process	8/9880	16/129511	7	1.8
GO:0008612	peptidyl-lysine modification to peptidyl-hypusine	8/9880	16/129511	9	1.8
GO:0006423	cysteinyl-tRNA aminoacylation	8/9880	16/129511	11	1.8
GO:0044267	cellular protein metabolic process	655/9880	6393/129511	5	1.79
GO:0044271	cellular nitrogen compound biosynthetic process	265/9880	2604/129511	4	1.79
GO:0006796	phosphate-containing compound metabolic process	506/9880	5361/129511	4	1.79
GO:0043412	macromolecule modification	672/9880	6051/129511	4	1.78
GO:0006139	nucleobase-containing compound metabolic process	688/9880	6267/129511	4	1.78
GO:0006777	Mo-molybdopterin cofactor biosynthetic process	13/9880	42/129511	7	1.77
GO:1901360	organic cyclic compound metabolic process	786/9880	6882/129511	3	1.77
GO:0006725	cellular aromatic compound metabolic process	753/9880	6732/129511	3	1.77
GO:0034641	cellular nitrogen compound metabolic process	877/9880	7987/129511	3	1.74
GO:0046483	heterocycle metabolic process	759/9880	6710/129511	3	1.74
GO:0010629	negative regulation of gene expression	30/9880	166/129511	6	1.71
GO:0016071	mRNA metabolic process	84/9880	674/129511	7	1.69
GO:0044260	cellular macromolecule metabolic process	972/9880	9893/129511	4	1.69
GO:1901564	organonitrogen compound metabolic process	1213/9880	12929/129511	3	1.63
GO:0043170	macromolecule metabolic process	1472/9880	15069/129511	3	1.62
GO:0016310	phosphorylation	341/9880	3524/129511	5	1.6
GO:0006807	nitrogen compound metabolic process	1772/9880	17828/129511	2	1.59
GO:0044237	cellular metabolic process	1906/9880	17064/129511	2	1.57
GO:0009098	leucine biosynthetic process	8/9880	17/129511	8	1.57
GO:0006435	threonyl-tRNA aminoacylation	8/9880	17/129511	11	1.57
GO:0044238	primary metabolic process	1962/9880	19530/129511	2	1.57
GO:0008152	metabolic process	2337/9880	22313/129511	1	1.56
GO:0071704	organic substance metabolic process	2192/9880	21160/129511	2	1.54
GO:0048519	negative regulation of biological process	57/9880	414/129511	3	1.53
GO:0033554	cellular response to stress	108/9880	929/129511	3	1.51
GO:0006325	chromatin organization	47/9880	323/129511	4	1.42
GO:1901136	carbohydrate derivative catabolic process	18/9880	79/129511	4	1.39
GO:0006950	response to stress	129/9880	1162/129511	2	1.38
GO:0042364	water-soluble vitamin biosynthetic process	28/9880	156/129511	5	1.37
GO:0019243	methylglyoxal catabolic process to D-lactate via S-lactoyl-glutathione	8/9880	18/129511	9	1.35
GO:0019310	inositol catabolic process	8/9880	18/129511	7	1.35
GO:0018344	protein geranylgeranylation	8/9880	18/129511	8	1.35
GO:0006566	threonine metabolic process	11/9880	34/129511	9	1.3

Table S8. enriched terms relating to biological processes in the positively selected animal necrotrophs.

go number	name	ratio in study	ratio in population	depth	−log10 of *p* value
GO:0006190	inosine salvage	8/7214	8/114793	11	4.22
GO:0032955	regulation of division septum assembly	6/7214	6/114793	8	3.9
GO:0036159	inner dynein arm assembly	6/7214	6/114793	8	3.9
GO:0046168	glycerol-3-phosphate catabolic process	12/7214	20/114793	7	3.7
GO:0015940	pantothenate biosynthetic process	15/7214	20/114793	8	3.7
GO:0072350	tricarboxylic acid metabolic process	13/7214	26/114793	6	3.41
GO:2000113	negative regulation of cellular macromolecule biosynthetic process	17/7214	50/114793	7	3.29
GO:0002098	tRNA wobble uridine modification	18/7214	50/114793	12	3.29
GO:0030488	tRNA methylation	15/7214	44/114793	11	3.24
GO:0051253	negative regulation of RNA metabolic process	17/7214	44/114793	7	3.24
GO:0003341	cilium movement	24/7214	57/114793	4	3.24
GO:0006536	glutamate metabolic process	15/7214	38/114793	9	3.23
GO:0009086	methionine biosynthetic process	14/7214	33/114793	10	3.11
GO:0006414	translational elongation	21/7214	80/114793	6	3.09
GO:0071897	DNA biosynthetic process	14/7214	39/114793	7	3.09
GO:1901031	regulation of response to reactive oxygen species	6/7214	7/114793	6	3.08
GO:0006425	glutaminyl-tRNA aminoacylation	6/7214	7/114793	11	3.08
GO:0051103	DNA ligation involved in DNA repair	6/7214	7/114793	8	3.08
GO:0032958	inositol phosphate biosynthetic process	13/7214	18/114793	7	3.05
GO:0001522	pseudouridine synthesis	29/7214	138/114793	8	3.03
GO:0006606	protein import into nucleus	24/7214	81/114793	10	3.0
GO:1901617	organic hydroxy compound biosynthetic process	19/7214	81/114793	4	3.0
GO:0017186	peptidyl-pyroglutamic acid biosynthetic process, using glutaminyl-peptide cyclotransferase	11/7214	14/114793	9	2.98
GO:0043648	dicarboxylic acid metabolic process	27/7214	105/114793	6	2.98
GO:0045892	negative regulation of transcription, DNA-templated	16/7214	40/114793	10	2.94
GO:0006525	arginine metabolic process	15/7214	53/114793	9	2.93
GO:0051056	regulation of small GTPase mediated signal transduction	27/7214	82/114793	7	2.92
GO:0009082	branched-chain amino acid biosynthetic process	24/7214	60/114793	5	2.91
GO:0016573	histone acetylation	21/7214	83/114793	11	2.83
GO:0019751	polyol metabolic process	19/7214	83/114793	5	2.83
GO:0009084	glutamine family amino acid biosynthetic process	22/7214	83/114793	9	2.83
GO:0007154	cell communication	18/7214	54/114793	2	2.82
GO:0006537	glutamate biosynthetic process	11/7214	19/114793	10	2.81
GO:0006166	purine ribonucleoside salvage	13/7214	19/114793	10	2.81
GO:0034637	cellular carbohydrate biosynthetic process	25/7214	133/114793	5	2.8
GO:0042398	cellular modified amino acid biosynthetic process	23/7214	116/114793	5	2.79
GO:0006066	alcohol metabolic process	26/7214	116/114793	4	2.79
GO:0017038	protein import	28/7214	116/114793	9	2.79
GO:0009064	glutamine family amino acid metabolic process	40/7214	142/114793	8	2.79
GO:0033014	tetrapyrrole biosynthetic process	21/7214	76/114793	5	2.78
GO:0001510	RNA methylation	40/7214	188/114793	8	2.78
GO:0009966	regulation of signal transduction	43/7214	245/114793	5	2.77
GO:0046034	ATP metabolic process	32/7214	198/114793	2	2.75
GO:0006096	glycolytic process	26/7214	134/114793	12	2.74
GO:0000398	mRNA splicing, via spliceosome	50/7214	286/114793	11	2.73
GO:0051274	beta-glucan biosynthetic process	17/7214	62/114793	8	2.71
GO:0016052	carbohydrate catabolic process	37/7214	236/114793	4	2.69
GO:0072330	monocarboxylic acid biosynthetic process	31/7214	101/114793	7	2.68
GO:0031324	negative regulation of cellular metabolic process	18/7214	70/114793	5	2.65
GO:0000097	sulfur amino acid biosynthetic process	20/7214	70/114793	5	2.65
GO:0032012	regulation of ARF protein signal transduction	16/7214	56/114793	9	2.6
GO:0060271	cilium assembly	27/7214	119/114793	7	2.59
GO:0008380	RNA splicing	53/7214	321/114793	8	2.59
GO:1901615	organic hydroxy compound metabolic process	37/7214	192/114793	3	2.58
GO:0046394	carboxylic acid biosynthetic process	96/7214	495/114793	6	2.53
GO:0006480	N-terminal protein amino acid methylation	6/7214	8/114793	9	2.52
GO:0070925	organelle assembly	35/7214	215/114793	5	2.52
GO:0018205	peptidyl-lysine modification	29/7214	165/114793	8	2.52
GO:0008033	tRNA processing	92/7214	407/114793	9	2.51
GO:0046129	purine ribonucleoside biosynthetic process	14/7214	50/114793	9	2.51
GO:0008652	cellular amino acid biosynthetic process	65/7214	388/114793	7	2.5
GO:0018193	peptidyl-amino acid modification	65/7214	497/114793	7	2.5
GO:0032787	monocarboxylic acid metabolic process	64/7214	357/114793	6	2.47
GO:0006400	tRNA modification	52/7214	220/114793	10	2.46
GO:0010605	negative regulation of macromolecule metabolic process	42/7214	220/114793	5	2.46
GO:0032259	methylation	64/7214	377/114793	2	2.46
GO:0016053	organic acid biosynthetic process	126/7214	602/114793	4	2.45
GO:0016071	mRNA metabolic process	80/7214	646/114793	7	2.45
GO:1901607	alpha-amino acid biosynthetic process	48/7214	329/114793	8	2.42
GO:0048583	regulation of response to stimulus	49/7214	258/114793	3	2.42
GO:1901605	alpha-amino acid metabolic process	71/7214	546/114793	7	2.4
GO:0042364	water-soluble vitamin biosynthetic process	24/7214	131/114793	5	2.39
GO:0043414	macromolecule methylation	48/7214	304/114793	5	2.38
GO:0009451	RNA modification	107/7214	512/114793	7	2.35
GO:0006165	nucleoside diphosphate phosphorylation	27/7214	159/114793	8	2.35
GO:0006397	mRNA processing	67/7214	528/114793	8	2.35
GO:0006399	tRNA metabolic process	132/7214	725/114793	8	2.35
GO:0034660	ncRNA metabolic process	167/7214	1014/114793	7	2.33
GO:0043604	amide biosynthetic process	110/7214	802/114793	5	2.32
GO:0007017	microtubule-based process	116/7214	1016/114793	2	2.32
GO:0006518	peptide metabolic process	111/7214	990/114793	5	2.31
GO:0034470	ncRNA processing	127/7214	690/114793	8	2.28
GO:0006412	translation	91/7214	714/114793	7	2.28
GO:0044283	small molecule biosynthetic process	158/7214	943/114793	3	2.27
GO:0072594	establishment of protein localization to organelle	35/7214	227/114793	6	2.27
GO:0006355	regulation of transcription, DNA-templated	112/7214	1004/114793	9	2.24
GO:0031323	regulation of cellular metabolic process	130/7214	1282/114793	4	2.21
GO:0010468	regulation of gene expression	143/7214	1284/114793	5	2.2
GO:0007018	microtubule-based movement	93/7214	774/114793	3	2.2
GO:1901575	organic substance catabolic process	147/7214	1452/114793	3	2.19
GO:0043603	cellular amide metabolic process	135/7214	1100/114793	4	2.19
GO:0034645	cellular macromolecule biosynthetic process	188/7214	1454/114793	5	2.19
GO:0006520	cellular amino acid metabolic process	132/7214	1045/114793	6	2.18
GO:0044262	cellular carbohydrate metabolic process	37/7214	258/114793	4	2.17
GO:0051171	regulation of nitrogen compound metabolic process	134/7214	1260/114793	4	2.17
GO:0006082	organic acid metabolic process	258/7214	1638/114793	3	2.17
GO:2000112	regulation of cellular macromolecule biosynthetic process	118/7214	1107/114793	6	2.16
GO:0051252	regulation of RNA metabolic process	113/7214	1021/114793	6	2.16
GO:0006396	RNA processing	240/7214	1505/114793	7	2.13
GO:0006091	generation of precursor metabolites and energy	38/7214	272/114793	3	2.12
GO:0009056	catabolic process	154/7214	1580/114793	2	2.12

Table S9. Enriched terms relating to biological processes in the positively selected animal necrotrophs (continued).

GO number	Name	Ratio in study	Ratio in population	Depth	−log10 of *p* value
GO:0006629	lipid metabolic process	143/7214	1475/114793	3	2.12
GO:0060255	regulation of macromolecule metabolic process	160/7214	1442/114793	4	2.11
GO:0019752	carboxylic acid metabolic process	216/7214	1483/114793	5	2.09
GO:0034613	cellular protein localization	36/7214	248/114793	4	2.08
GO:1901566	organonitrogen compound biosynthetic process	277/7214	2065/114793	4	2.08
GO:0009059	macromolecule biosynthetic process	189/7214	1710/114793	4	2.07
GO:0006102	isocitrate metabolic process	7/7214	13/114793	7	2.05
GO:0050789	regulation of biological process	306/7214	3078/114793	2	2.03
GO:0016043	cellular component organization	184/7214	2008/114793	3	2.02
GO:0044281	small molecule metabolic process	317/7214	2782/114793	2	2.01
GO:0006401	RNA catabolic process	29/7214	185/114793	7	2.01
GO:0044271	cellular nitrogen compound biosynthetic process	215/7214	2275/114793	4	2.0
GO:0016070	RNA metabolic process	372/7214	2742/114793	6	2.0
GO:0050794	regulation of cellular process	270/7214	2861/114793	3	1.97
GO:0071840	cellular component organization or biogenesis	189/7214	2119/114793	2	1.96
GO:0090304	nucleic acid metabolic process	466/7214	3855/114793	5	1.96
GO:0044249	cellular biosynthetic process	430/7214	3741/114793	3	1.96
GO:1901576	organic substance biosynthetic process	464/7214	4002/114793	3	1.93
GO:0065007	biological regulation	330/7214	3366/114793	1	1.93
GO:0006139	nucleobase-containing compound metabolic process	548/7214	4921/114793	4	1.92
GO:0009058	biosynthetic process	506/7214	4268/114793	2	1.91
GO:0051273	beta-glucan metabolic process	22/7214	122/114793	7	1.88
GO:0006725	cellular aromatic compound metabolic process	604/7214	5329/114793	3	1.87
GO:1901360	organic cyclic compound metabolic process	632/7214	5489/114793	3	1.86
GO:0043412	macromolecule modification	580/7214	6499/114793	4	1.86
GO:0006555	methionine metabolic process	15/7214	64/114793	9	1.85
GO:0034641	cellular nitrogen compound metabolic process	717/7214	6287/114793	3	1.84
GO:0006464	cellular protein modification process	469/7214	5979/114793	6	1.83
GO:0046483	heterocycle metabolic process	600/7214	5319/114793	3	1.82
GO:0022607	cellular component assembly	90/7214	869/114793	4	1.81
GO:0044267	cellular protein metabolic process	566/7214	6741/114793	5	1.81
GO:0006793	phosphorus metabolic process	450/7214	5743/114793	3	1.78
GO:0019538	protein metabolic process	725/7214	9516/114793	4	1.77
GO:0044260	cellular macromolecule metabolic process	824/7214	9171/114793	4	1.74
GO:0006310	DNA recombination	31/7214	211/114793	7	1.7
GO:0044237	cellular metabolic process	1576/7214	15465/114793	2	1.67
GO:1901564	organonitrogen compound metabolic process	1025/7214	12079/114793	3	1.67
GO:0043170	macromolecule metabolic process	1243/7214	13653/114793	3	1.66
GO:0006541	glutamine metabolic process	10/7214	31/114793	9	1.65
GO:0006418	tRNA aminoacylation for protein translation	40/7214	302/114793	10	1.64
GO:0005975	carbohydrate metabolic process	121/7214	1279/114793	3	1.63
GO:0006796	phosphate-containing compound metabolic process	444/7214	5726/114793	4	1.63
GO:0006807	nitrogen compound metabolic process	1481/7214	15957/114793	2	1.63
GO:0044238	primary metabolic process	1583/7214	17428/114793	2	1.62
GO:0008152	metabolic process	1908/7214	19763/114793	1	1.61
GO:0071704	organic substance metabolic process	1793/7214	18791/114793	2	1.59
GO:0009987	cellular process	2251/7214	23958/114793	1	1.56
GO:0008150	biological_process	2760/7214	30756/114793	0	1.53
GO:0000096	sulfur amino acid metabolic process	22/7214	130/114793	4	1.43
GO:0002943	tRNA dihydrouridine synthesis	6/7214	11/114793	11	1.41
GO:0009072	aromatic amino acid family metabolic process	26/7214	169/114793	4	1.4

Table S10. Significant enriched terms relating to biological processes in the stramenopile dataset's paralogs.

GO number	Name	Ratio in study	Ratio in population	Depth	−log10 *p* value	Species
GO:0055085	transmembrane transport	16/62	557/11080	4	3.26	Achlya hypogyna
GO:0019637	organophosphate metabolic process	8/62	213/11080	4	1.36	Achlya hypogyna
GO:0043412	macromolecule modification	107/760	755/8600	4	2.26	Albugo candida
GO:0071704	organic substance metabolic process	271/760	2317/8600	2	2.12	Albugo candida
GO:0044237	cellular metabolic process	251/760	2142/8600	2	2.09	Albugo candida
GO:0044238	primary metabolic process	254/760	2154/8600	2	2.07	Albugo candida
GO:0006464	cellular protein modification process	95/760	671/8600	6	2.06	Albugo candida
GO:0008152	metabolic process	283/760	2451/8600	1	2.04	Albugo candida
GO:0044260	cellular macromolecule metabolic process	145/760	1177/8600	4	1.58	Albugo candida
GO:0043170	macromolecule metabolic process	196/760	1696/8600	3	1.45	Albugo candida
GO:0006807	nitrogen compound metabolic process	223/760	1970/8600	2	1.44	Albugo candida
GO:0006796	phosphate-containing compound metabolic process	90/760	660/8600	4	1.41	Albugo candida
GO:0044262	cellular carbohydrate metabolic process	12/504	37/8647	4	2.94	Albugo laibachii
GO:0034645	cellular macromolecule biosynthetic process	35/504	228/8647	5	2.47	Albugo laibachii
GO:0051560	mitochondrial calcium ion homeostasis	4/504	4/8647	10	1.7	Albugo laibachii
GO:0042592	homeostatic process	8/504	21/8647	3	1.64	Albugo laibachii
GO:0051274	beta-glucan biosynthetic process	6/504	11/8647	8	1.62	Albugo laibachii
GO:0098771	inorganic ion homeostasis	6/504	12/8647	6	1.34	Albugo laibachii
GO:0034637	cellular carbohydrate biosynthetic process	8/504	23/8647	5	1.31	Albugo laibachii
GO:0006821	chloride transport	6/477	22/17944	7	1.48	Aphanomyces astaci
GO:0045048	protein insertion into ER membrane	4/140	5/14252	8	4.08	Aphanomyces euteiches
GO:0046434	organophosphate catabolic process	4/140	16/14252	5	1.56	Aphanomyces euteiches
GO:0009084	glutamine family amino acid biosynthetic process	8/272	19/6501	9	3.14	Bremia lactucae
GO:0006561	proline biosynthetic process	6/272	10/6501	10	2.81	Bremia lactucae
GO:1901264	carbohydrate derivative transport	6/272	13/6501	7	1.94	Bremia lactucae
GO:0044271	cellular nitrogen compound biosynthetic process	0/272	274/6501	4	1.59	Bremia lactucae
GO:0006259	DNA metabolic process	18/843	803/12755	6	2.4	Globisporangium splendens
GO:0015074	DNA integration	2/843	666/12755	7	2.31	Globisporangium splendens
GO:0044260	cellular macromolecule metabolic process	81/843	1879/12755	4	1.68	Globisporangium splendens
GO:0034220	ion transmembrane transport	12/197	68/7213	5	3.39	Hyaloperonospora arabidopsidis
GO:0098656	anion transmembrane transport	8/197	33/7213	6	2.45	Hyaloperonospora arabidopsidis
GO:0055085	transmembrane transport	20/197	247/7213	4	1.69	Hyaloperonospora arabidopsidis
GO:0043933	protein-containing complex subunit organization	44/3329	134/20260	4	2.26	Nothophytophthora sp
GO:1901564	organonitrogen compound metabolic process	379/3329	2777/20260	3	1.4	Nothophytophthora sp
GO:0006665	sphingolipid metabolic process	4/123	20/13965	6	1.32	Phytophthora cinnamomi
GO:0007186	G protein-coupled receptor signaling pathway	4/201	12/19214	5	1.98	Phytophthora fragariae
GO:0098771	inorganic ion homeostasis	4/201	13/19214	6	1.83	Phytophthora fragariae
GO:0090304	nucleic acid metabolic process	2/201	1600/19214	5	1.81	Phytophthora fragariae
GO:1901360	organic cyclic compound metabolic process	4/201	1865/19214	3	1.4	Phytophthora fragariae
GO:0034219	carbohydrate transmembrane transport	2/41	2/8291	8	1.37	Phytophthora kernoviae
GO:0006643	membrane lipid metabolic process	6/103	37/18043	5	3.97	Phytophthora megakarya
GO:0009247	glycolipid biosynthetic process	4/103	18/18043	7	2.29	Phytophthora megakarya
GO:0006470	protein dephosphorylation	18/1465	55/12653	7	1.3	Phytophthora nicotianae
GO:0070536	protein K63-linked deubiquitination	4/748	5/17216	10	1.48	Phytophthora parasitica
GO:0006069	ethanol oxidation	2/43	2/9298	7	1.41	Pythium aphanidermatum
